# Comparing substance use and mental health among sexual and gender minority and heterosexual cisgender youth experiencing homelessness

**DOI:** 10.1371/journal.pone.0248077

**Published:** 2021-03-11

**Authors:** Jennifer Hao, Matthew Beld, Ladan Khoddam-Khorasani, Annesa Flentje, Eva Kersey, Haley Mousseau, Julie Frank, Adam Leonard, Sebastian Kevany, Carol Dawson-Rose

**Affiliations:** 1 University of California, San Francisco (UCSF) School of Medicine, San Francisco, CA, United States of America; 2 University of California, San Francisco (UCSF) School of Nursing, San Francisco, CA, United States of America; 3 Larkin Street Youth Services, San Francisco, CA, United States of America; 4 Department of HIV/AIDS, University of California, San Francisco (UCSF), CA, United States of America; Hunter College, UNITED STATES

## Abstract

Youth homelessness has been demonstrated to disproportionately affect sexual and gender minority (SGM) youth compared to heterosexual cisgender peers. In this context, we aimed to compare health risks between service-seeking SGM and heterosexual cisgender youth experiencing homelessness, including harmful risks stemming from substance use and severity of symptoms of mental health disorders. We recruited 100 racially diverse, unstably housed participants aged 18–24 who access services at an urban non-profit organization in San Francisco, CA. Data analysis included 56 SGM participants who identified as gay, lesbian, bisexual, pansexual, unsure, transgender, and nongender, and 44 heterosexual cisgender participants. In contrast to previous studies reporting significantly higher frequency of substance use and more severe symptoms of depression, generalized anxiety, and post-traumatic stress disorder among SGM youth compared to heterosexual cisgender peers, many of these health disparities were not observed in our diverse study population of service-seeking youth. Furthermore, with the exception of methamphetamine, SGM participants did not exhibit greater harmful risks resulting from substance use, such as health, social, financial, and legal complications. We discuss the reduced burden of health disparities between SGM and heterosexual cisgender youth in our service-seeking study population within the context of gender- and sexuality-affirming programming offered at the partnering community organization. We conclude that longitudinal data on these tailored community-level interventions are needed to further explore the reduced burden of health disparities observed among service-seeking SGM youth experiencing homelessness in San Francisco in order to continue supporting pathways out of homelessness for youth of all sexual and gender identities nationwide.

## Introduction

It is estimated that homelessness affects 3.5 million youth between the ages of 18 to 25 annually in the United States [1]. Sexual and gender minority (SGM) youth are overrepresented in homeless populations, with research indicating that between 30% and 40% of service-using homeless youth identify as SGM, within the context of approximately 6.4% of youth aged 18 to 29 identifying as SGM nationwide [2,3]. SGM youth include individuals who identify as lesbian, gay, bisexual, and transgender (LGBT) as well as genderqueer, non-binary, agender, asexual, or another sexual or gender identity that is either or both non-heterosexual or non-cisgender (defined as someone whose gender aligns with traditional norms related to the sex they were assigned at birth). Previous studies examining pathways into homelessness among youth have repeatedly demonstrated that SGM youth are more likely to enter homelessness as a result of family members who are unaccepting of their gender identity and sexuality compared to heterosexual cisgender peers, demonstrating how SGM status is itself a risk factor for becoming homeless [1].

In addition to disproportionately high representation among all unstably housed youth, SGM youth experiencing homelessness also face increased health risks compared to their heterosexual cisgender peers. With regards to mental health, lesbian, gay, bisexual, and transgender (LGBT) youth who are homeless are more likely to experience substance use and use a greater number of substances than heterosexual cisgender peers experiencing homelessness [2–4]. Given these documented disparities, SGM homeless youth may be at higher risk for negative health outcomes related to substance use, such as HIV and viral hepatitis, which can further serve as a barrier to maintaining stable housing [5]. Sexual and gender minority youth experiencing homelessness also report worse mental health outcomes, including increased suicidal ideation and more severe depressive symptoms [6], increased anxiety [7], and higher rates of post-traumatic stress disorder (PTSD) [8].

One proposed model for conceptualizing these observed health disparities among SGM youth is the minority stress model, which acknowledges that SGM communities face an excessive burden of daily stigma and discrimination from living in a heterosexist, transphobic society, resulting in detrimental effects to their emotional, psychological, and physical health [9]. These experiences of stigma and discrimination among SGM youth have been documented to occur in a wide variety of settings, including family rejection, homophobic bullying in community settings such as schools [10], and discrimination from clients and staff in emergency shelters [11].

In addition to the violence and discrimination faced due to their sexual orientation or gender identity, SGM youth experiencing homelessness often hold multiple identities that place them into further marginalized groups, such as their racial and ethnic backgrounds. Youth of color, particularly Black youth, are more likely to experience homelessness compared to white peers [12]. Furthermore, SGM youth of color face more difficult exits from homelessness compared to their white, heterosexual, cisgender peers [13]. Black LGBT youth experiencing homelessness are more likely to experience harassment from police and community members, as well as increased sexualization and invisibility [14], which collectively make LGBT youth of color more vulnerable to various mental health disparities, such as increased substance use and prevalence of mood disorders.

Policy agendas aimed at addressing the health disparities faced by SGM youth experiencing homelessness have emphasized the need to understand SGM youth as non-homogenous micro-communities with unique experiences, risk factors, and social environments [15]. Previous studies of youth experiencing homelessness in San Francisco, the location of this study, have revealed high burdens of substance use and mental health conditions in line with national trends. In the *San Francisco 2019 Homeless Unique Youth Count & Survey*, one in five homeless individuals on a single night was under the age of 25 [16]. Of these unstably housed youth, nearly half (46%) identified as LGBTQ+. One in three surveyed youth reported ongoing drug or alcohol use, and 13% reported substance use as a cause of their homelessness. Mental health was another commonly reported cause of homelessness, with 30% of all San Francisco homeless youth indicating that their mental health was a contributing cause of homelessness [16]. Symptoms of depression, PTSD, and anxiety among service-seeking San Francisco youth experiencing homelessness are correlated with increased prevalence of opioid and stimulant use, demonstrating the inter-connectedness of substance use and mental health outcomes [17]. The high health burden of substance use and mood disorders is also tied to increased mortality among San Francisco youth experiencing homelessness, who experience mortality rates 10 times in excess of their stably housed, age-matched peers, with a majority of deaths resulting from substance use or suicide [18].

While the disparities in the prevalence of substance use and negative mental health outcomes among SGM youth experiencing homelessness compared to heterosexual cisgender peers are well-described in the literature, a comparative understanding of downstream harms (such as substance dependence and health, social, financial, and legal complications) associated with use of specific substances between SGM and heterosexual cisgender youth experiencing homelessness remains poorly characterized. In this study, we employ a tool that quantifies the burden of negative impacts associated with use of a specific substance. Using a cross-sectional analysis of a racially diverse group of service-seeking youth experiencing homelessness ages 18 to 24 in a dense, urban environment, we examine risks of harmful use associated with specific substances among SGM youth experiencing homelessness compared to their heterosexual cisgender peers. Alongside substance use, we examined whether symptoms of depression, anxiety, and PTSD differed between SGM and heterosexual cisgender youth who sought services at a community-based site. We expected that SGM youth would exhibit greater health risks associated with substance use across all substances surveyed, as well as more severe symptoms of mental illness when compared with their heterosexual cisgender peers. All data were collected from a capacity-building initiative at a partnering multi-site, non-profit community-based organization in San Francisco, California.

## Materials and methods

### Design, sample, setting and interviews

Our study was designed as a cross-sectional investigation of youth aged 18 to 24 who utilized services from Larkin Street Youth Services, a community-based organization (CBO) in San Francisco. Each year, Larkin Street serves 2,500 to 3,000 youth aged 12 to 24 years old. Clients include individuals who live outside or in a car, a shelter, a transitional living program, permanent supportive housing, a single-room occupancy hotel, a unit partially paid for using subsidies, or who are otherwise unstably housed. The organization also offers a wide array of programming, including emergency and transitional housing, basic needs services such as access to food, showers, laundry, and harm reduction supplies, educational and employment training programs, medical care, behavioral health services, case management, street outreach, and a youth leadership development program. Larkin Street also offers resources and programming specifically for SGM youth, and staff members undergo LGBTQ cultural competency trainings.

In order for participants to qualify for our survey, they needed to (1) be between 18 and 24 years of age and (2) utilize services at Larkin Street. Recruitment strategies included posting flyers within the CBO’s residential and clinical spaces, referrals of participants from frontline workers, case managers, counselors, and group facilitators, and presentations at community housing meetings. Of note, a small subset of participants (*n =* 13) were surveyed at a service site that only serves youth living with a HIV diagnosis, but the majority of participants (*n =* 87) were recruited from Larkin Street’s other sites that serve all youth, including daytime drop-in centers, health clinics, and transitional housing spaces.

### Questionnaire design and administration

Our research team conducted 100 interviews of youth aged 18 to 24 between May 2017 and April 2018, with written informed consent obtained before each interview. Our survey questionnaire was composed of the Government Performance and Results Act (GPRA) survey for adolescents, supplemented with validated measures for substance use, anxiety, depression, PTSD, adverse childhood experiences, and quality of life. Based on consultations with frontline service delivery staff at Larkin Street, it was considered most effective for the interviewer to sit with the participant while they were completing the questionnaire in order to answer any questions the participant might have. If the participant preferred to complete the questionnaire in private, the research team member left the interview room. All participants who initiated the survey were provided with a $30 gift card to thank them for participating in the survey. Participation in the questionnaire had no bearing on individuals’ ability to obtain services at Larkin Street. All of the above stages of data collection were approved by the University of California, San Francisco’s (UCSF) Institutional Review Board (Committee on Human Research).

#### Data collection tools

*Demographics*. Participants reported on age, race, ethnicity, and highest level of education attained (Table 1). To determine sexual orientation and gender identity, participants also reported on sex assigned at birth, gender identity, and sexual orientation in multiple-choice and open-field format. Options for gender identity included “male”, “female”, and “other”, which allowed participants to write answers into an open-field option. Options for sexual orientation included “straight or heterosexual”, “bisexual”, “gay or lesbian”, and “unsure”.

**Table 1:** Description of study participant characteristics by SGM status.

Characteristic	Total	SGM*	Heterosexual Cisgender	
	*n* = 100	*n =* 56 (%)	*n* = 44 (%)	*p-value*
**Age (Mean ± SD)**	21.7 ± 1.8	21.6 ± 1.9	22.0 ± 1.7	0.24
**Gender Identity**				0.08
Male	67	38 (68)	29 (66)	
Female	28	13 (25)	15 (34)	
Other	5	5 (6)	0	
**Race**				0.08
Mixed Race	38	24 (43)	14 (32)	
Black/African-American	28	10 (19)	18 (42)	
White	22	15 (28)	7 (16)	
Other	12	7 (13)	5 (12)	
**Highest Level of Education**				0.75
Elementary School	1	1 (2)	0	
Middle School	8	4 (7)	4 (9)	
High School	69	37 (66)	32 (73)	
Beyond High School	22	14 (25)	8 (18)	
**Describe Where You Live**				0.09
Homeless or in a shelter	50	26 (46)	24 (55)	
Other	18	14 (25)	4 (9)	
In my own home or apartment	15	8 (14)	7 (16)	
In a group home	9	6 (11)	3 (7)	
In a relative’s home	4	0	4 (9)	
In campus/dormitory housing	4	2 (4)	2 (5)	
**HIV Status**				< 0.01
HIV-	65	28 (55)	37 (97)	
HIV+	23	22 (43)	1 (3)	
Don’t Know/Unsure	1	1 (2)	0	
**Used Substance In Last 3 months**				
Street Opioids	12	5 (9)	7 (16)	0.22
Methamphetamines	28	18 (32)	10 (23)	0.3
Cannabis	46	28 (50)	18 (41)	0.37
Cocaine	32	20 (36)	12 (27)	0.37
Sedatives	19	13 (23)	6 (14)	0.23
Hallucinogens	25	17 (30)	8 (18)	0.16
Prescription Opioids	13	5 (9)	8 (18)	0.14
Inhalants	7	5 (9)	2 (5)	0.33
Prescription Stimulants	10	5 (9)	5 (11)	0.47

*Included in the SGM category are: Gay or Lesbian (n = 26), Bisexual (n = 25), Unsure (n = 4), Pansexual (n = 1), Transgender (n = 3), Nongender (n = 1).

*Government Performance and Results Act (GPRA) Questionnaire*. Per federal mandate, all Substance Abuse and Mental Health Services Administration (SAMHSA) grantees are required to collect and report performance data using the GPRA questionnaire. Data collection for GPRA includes measures on demographics, substance use, and HIV infection. To assess HIV status, participants were asked (1) if they have ever been tested for HIV and (2) to estimate the date of their last HIV test and (3) to self-identify their HIV status. Of note, concomitant testing was not performed to confirm self-reported serostatus.

*National Institute on Drug Abuse-Modified Alcohol*, *Smoking*, *and Substance Involvement Screening Test V2*.*0 (NIDA-Modified ASSIST)*. The NIDA-Modified ASSIST V2.0 is a screening test for individuals who may have been, or are currently, at risk of developing a substance use disorder. The ASSIST questionnaire assesses for use of tobacco, alcohol, cannabis, cocaine, stimulants, sedatives, hallucinogens, inhalants, opioids, and other drugs. Individuals are asked to report whether or not they have ever used each substance in their lifetime, and then how frequently they used each substance within the past three months. For each substance used in the past 3 months, the respondent then answers five additional questions to measure harmful use associated with each substance. Q3 asks about compulsion (a measure of psychological dependence), Q4 asks about health, social, financial, or legal complications associated with substance use, Q5 asks whether participants have failed to fill responsibilities of a usual role due to use of that substance, Q6 asks whether close contacts have expressed concern about their substance use, and Q7 asks about prior failed attempts to cut down on substance use. Based on responses to these latter questions measuring harmful use, a Substance Involvement (SI) score can be calculated that correlates to the overall health risk level associated with use of each specific substance. An SI score of 0 to 3 is considered low risk of harmful use, 4 to 26 equates to moderate risk, and 27+ indicates high risk of harmful use. The tool is intended to help clinicians assess the level of health-related risks associated with an individual’s substance use habits [19]. The NIDA ASSIST has been validated for use in both adolescents and adults, including individuals who use a number of substances and exhibit varying degrees of use across different substances [20,21].

*Center for Epidemiologic Studies-Depression (CES-D)*. The *CES-D* is a 20-item validated measure that asks participants to describe symptoms that have been associated with depression over the past week. Scores range from 0 to 60, with higher scores indicating greater depressive symptoms. A score greater than 15 indicates increased risk for clinical depression [22].

*PTSD Checklist for DSM-5 (PCL-5)*. The *PCL-5* is a 20-item validated measure that screens for the presence of symptoms of post-traumatic stress disorder (PTSD). Participants are asked to consider “a very stressful experience” and answer questions about symptoms of PTSD over the past month specifically in response to the stressful experience. Scores range from 0 to 80, with higher scores indicating more severe symptoms of PTSD. A PCL-5 cut-off score of 33 can be used to screen for symptoms consistent with clinical PTSD [23].

*Generalized Anxiety Disorder instrument (GAD-7)*. The *GAD-*7 is a 7-question validated tool to assess symptoms of generalized anxiety. Participants are asked to respond to questions regarding different symptoms of anxiety over the past 2 weeks. A total score of 10 or above on a scale of 0 to 21 indicates symptoms of moderate anxiety, with increasing scores indicating greater functional impairment due to clinical symptoms [24].

### Data analysis

The research team conducted a total of 103 interviews; three participants were unable to complete the entirety of the questionnaire and their data was excluded from analysis. Our analysis defined homelessness as (1) living in a supervised publicly- or privately-operated shelter designated to provide temporary living arrangement or (2) living within a primary nighttime residence that is a public or private place not designed for (or ordinarily used) as a regular sleeping accommodation including car parks, abandoned buildings, bus or train stations, airports, and camping grounds [16]. Participants who identified as gay, lesbian, bisexual, unsure, or self-identified in write-in responses as “pansexual”, “transgender”, and “nongender” were categorized as SGM youth in data analysis.

Pearson’s Chi-square analysis, Fisher’s Exact Test, and independent t-tests were used to test associations between study participant demographics and anxiety, depression, PTSD, and SGM status. One-tailed independent t-tests were used to test associations between mean ASSIST scores by substance and SGM status, with the hypothesis that SGM youth would exhibit higher levels of substance use and associated risks than heterosexual cisgender participants. All analyses were conducted using STATA Version 14.2.

## Results

### Demographics

Of the 100 participants included in the analysis, the mean age was 21.7 years, with an age range from 18 to 24 years. Of these, 67% of participants identified as male, 28% as female, and 5% as another gender identity. 52% of participants identified as lesbian, gay, or bisexual. More than three-quarters of participants (77%) were people of color, including participants who identified as Black, American Indian or Alaska Native, Filipino, Pacific Islander, or Multiracial (identifying as more than one race), which correlates closely to the overall demographics of youth served at Larkin Street [25]. With regards to education, 91% of participants had completed at least a high school education. Overall, 23% of surveyed participants self-reported living with an HIV diagnosis, including 43% of surveyed SGM youth, compared to only 3% of their heterosexual cisgender peers (p < 0.01) (Table 1).

### Substance use and risk

Data on current or recent substance use revealed that 35 SGM participants (55%) and 21 heterosexual cisgender participants (48%) had used at least one substance in the past three months. The four most commonly used substances among all 100 participants were cannabis (46%), cocaine and crack cocaine (32%), methamphetamine (28%) and hallucinogens (25%). Of note, recent use within the past 3 months did not differ significantly by SGM status for any of the substance types surveyed (Table 1).

Using the NIDA-modified ASSIST tool, we also assessed whether service-seeking SGM youth reported higher risk levels for negative health outcomes from substance use. Across all nine substances surveyed, a significant difference in ASSIST SI scores by SGM status was only noted for methamphetamine use (Table 2). Using the ASSIST tool, SGM participants reported higher mean health risk scores associated with methamphetamine use compared to heterosexual cisgender participants (8.1 vs 4.4; p = 0.05). For the eight other substances analyzed, no significant increase in health risks scores were observed among service-seeking SGM participants compared to heterosexual cisgender participants (Table 2).

**Table 2:** Mean substance involvement scores among participants from the National Institute on Drug Abuse-Modified Alcohol, smoking, and substance involvement screening test (*n* = 100).

* *	Total*	SGM	Heterosexual Cisgender	
Substance	*Mean (SD)*	*Mean (SD)*	*Mean (SD)*	*p-value*
**Street Opioids**	3.5 (10.1)	2.9 (9.1)	4.3 (11.3)	0.74
**Methamphetamines**	6.5 (11.1)	8.1 (12.3)	4.4 (9.2)	0.05
**Cannabis**	7.6 (9.8)	7.6 (9.0)	7.7 (4.4)	0.52
**Cocaine**	4.1 (8.6)	2.6 (4.9)	6.0 (11.4)	0.96
**Sedatives**	2.1 (5.2)	2.7 (5.7)	1.4 (4.4)	0.11
**Hallucinogens**	2.6 (5.5)	3.1 (5.8)	2.0 (5.1)	0.15
**Prescription Opioids**	1.4 (3.6)	1.0 (2.8)	1.8 (4.5)	0.99
**Inhalants**	0.7 (2.4)	0.9 (3.0)	0.3 (1.3)	0.11
**Prescription Stimulants**	0.8 (2.2)	1.0 (2.6)	0.5 (1.7)	0.13

*Scores of 0–3 = low risk of harmful use, 4–26 = moderate risk, and 27+ = high risk of harmful use.

### Mental health

In addition to assessing substance use, mental health measures were also analyzed by SGM identity. Overall, service-seeking SGM youth were noted to experience more severe symptoms of generalized anxiety compared to their heterosexual cisgender peers (p = 0.03). On average, SGM youth scored 10.8 on the GAD-7 –a score which correlates to moderate clinical symptoms of anxiety–whereas heterosexual cisgender youth scored an average of 8.5, which correlates to mild symptoms of anxiety. Of note, nearly one-third (32%) of SGM youth had severe-range anxiety symptoms, compared to only 16% of heterosexual cisgender youth (Table 3).

**Table 3:** Associations between SGM status and symptoms of generalized anxiety, PTSD, and depression among study participants (*n* = 99).

	Total	SGM	Heterosexual Cisgender	
	*Mean (%)*	*Mean (%)*	*Mean (%)*	*p-value *
GAD-7 (Anxiety)				0.07
***Mean Score ± SD***	9.8 ± 6.3	10.8 ± 6.2	8.5 ± 6.2	0.03
***Minimal (Score 0–4)***	23 (23)	8 (14)	15 (35)	
***Mild (Score 5–9)***	26 (26)	16 (29)	10 (23)	
***Moderate (Score 10–14)***	25 (25)	14 (25)	11 (26)	
***Severe (Score 15+)***	25 (25)	18 (32)	7 (16)	
PCL-5 (PTSD)				0.51
***Mean Score ± SD***	54.7 ± 21.9	57.2 ± 21.5	51.3 ± 22.3	0.10
***Score < 33***	20 (20)	10 (18)	10 (23)	
***Score ≥ 33***	79 (80)	46 (82)	33 (77)	
CES-D (Depression)				0.22
***Mean Score ± SD***	25.3 ± 12.3	26.7 ± 12.3	23.6 ± 12.3	0.10
***Score < 16***	26 (26)	12 (21)	14 (33)	
***Score ≥ 16***	73 (74)	44 (79)	29 (67)	

Notably, with respect to PTSD, no significant differences were found between PCL-5 scores for symptoms of PTSD between service-seeking SGM and heterosexual cisgender youth. However, high rates of PTSD symptoms were seen among participants overall, with 80% of participants with PCL-5 scores screening positive for symptoms of PTSD (Table 3). Similarly, the CES-D depression screening tool revealed that 74% of participants scored at or above 16 (the cutoff for identifying individuals at risk of clinical depression), although similarly high rates were found among both SGM youth and their heterosexual cisgender peers (Table 3). All mental health validated questionnaires demonstrated strong internal consistency among our study population, with Cronbach’s alpha values for the GAD-7, PCL-5, and CES-D of 0.90, 0.96, and 0.83, respectively.

## Discussion

In our study, service-seeking SGM youth experiencing homelessness did not exhibit higher levels of recent substance use within the past three months compared to their heterosexual cisgender peers. Across all nine substances analyzed, SGM and heterosexual cisgender participants were equally likely to have reported use of the substance in question at least once within the past 3 months. This finding differs from several existing studies which reported higher levels of substance use among SGM youth experiencing homelessness [2–4]. Beyond strictly measuring frequency of recent use, our study also demonstrated that compared to heterosexual cisgender youth, service-seeking SGM youth reported comparable risk levels of harmful outcomes (such as psychological dependence, and health, financial or legal implications) associated with substance use for all substances measured, with the exception of methamphetamine.

In regards to methamphetamine use, our finding that SGM participants reported higher associated risk levels fits into the well-established body of literature describing the high prevalence and associated risks of methamphetamine among gay and bisexual men [26]. Additionally, previous research has demonstrated that methamphetamine use disorder is uniquely difficult to treat, though contingency management programs provide the best evidence for efficacy [27]. Thus, the increased risk specifically associated with methamphetamine use among SGM youth identified in our study may be an important indicator that SGM youth require more tailored interventions for substance use disorders compared to the general homeless youth population.

It is unclear why our study did not find significant differences in frequency and harmful risks of substance use among SGM youth experiencing homelessness and their heterosexual cisgender counterparts, though several possibilities exist. One possible explanation is the relatively high availability of supportive services and communities for SGM youth in San Francisco. In a qualitative study of gay and transgender youth of color in San Francisco, participants described the Castro, a historically queer neighborhood of San Francisco, as a place “where they each spent time and sought safety, community, and identification” [14]. Larkin Street, our partnering community organization, provides transitional housing in the Castro for clients who identify as LGBTQ, including housing specifically for transgender youth experiencing homelessness. While programs in other cities may similarly support the needs of youth experiencing homelessness, they may also be settings in which SGM youth experience discrimination. For example, homeless youth in Canada have reported experiences of discrimination due to their SGM status from both other clients and staff in emergency shelters [11].

The relatively higher availability of SGM-friendly services for unstably housed youth in San Francisco, including our partnering organization Larkin Street Youth Services, may in turn have helped mitigate the underlying factors leading to higher prevalence and risk of substance use and mental health symptoms among SGM youth described in other studies, such as discrimination and stigma. For example, staff at Larkin Street create a welcoming environment for all youth by using SGM-supportive language and imagery on program materials. Additionally, all staff undergo LGBTQ cultural competency training, a core tenant of public health policy recommendations aimed at improving outcomes for SGM youth experiencing homelessness [15]. Available resources at Larkin Street specifically for SGM youth include culturally competent case management, aforementioned housing in neighborhoods that are welcoming to LGBTQ+ residents, and SGM-specific services such as support with official name and gender changes, and providing material needs such as chest binders. Larkin Street also offers substance use interventions such as harm reduction supplies and support groups to help individuals manage the intersection of substance use, trauma, and PTSD.

The availability of these services may have been especially important among SGM participants within our study population, as previous studies have shown that SGM youth experiencing homelessness are more likely to utilize service programs, such as STI and HIV testing, food access, street outreach teams, and counseling services compared to their heterosexual cisgender peers [28]. Though the proportion of SGM study participants who utilized the specific services described above is unknown, all study participants were recruited directly through Larkin Street. Thus, the availability and use of such services by our sample of service-seeking participants may have represented a de facto substance use intervention that minimized the harmful risks of substance use observed among SGM youth in other studies with greater proportions of non-service-seeking youth.

Among mental health symptoms, our study found a significant difference in generalized anxiety symptoms between SGM and heterosexual cisgender youth. However, this difference was not observed for symptoms of depression and PTSD, which stands in contrast to the previous literature demonstrating that SGM homeless youth are more likely to experience more severe symptoms of both depression and PTSD compared to heterosexual cisgender youth experiencing homelessness [2,7,8,29]. Reviewed collectively, our findings suggest that the minority stress experienced by unstably housed SGM youth increases their burden of day-to-day generalized anxiety, separate and in addition to the anxiety stemming from post-traumatic stress.

Lastly, given the overrepresentation of Black and Hispanic individuals among youth experiencing homelessness nationwide [1], the racial diversity of our sample is an important strength to highlight: 78% of total participants identified as a person of color, and 73% of SGM participants identified as a person of color. Given the increased victimization, invisibility, and more difficult exits from homelessness faced by SGM youth of color, it is critically important to capture the experiences of these communities to address the disproportionate risks of homelessness they face.

### Limitations

Because our sample was comprised of youth experiencing homelessness who already access non-profit services, our sample may have missed more extreme outlier cases related to mental health and substance use disorders and may have limited generalizability to youth experiencing homelessness who do not access services. In relation to mental health, our PTSD measure potentially overestimates the prevalence of PTSD among our study population, as some symptoms of PTSD (such as constant vigilance and difficulty with sleep) may be related to housing instability rather than strictly symptoms of PTSD. Additionally, our participants were often exposed to a multitude of traumatic stressors, and we were unable to isolate the specific event connected to symptoms of PTSD, as participants were not asked to describe their traumatic experiences. In terms of data analysis, our study was a relatively small sample of youth experiencing homelessness compared to other studies; as a result, the power to find associations by SGM status may be limited. In particular, the number of transgender and gender minority youth in our sample was relatively small compared to sexual minority participants, which may limit generalizability of our findings to service-seeking transgender youth experiencing homelessness. Third, our participants were all ages 18 to 24, which may reduce generalizability of our findings to youth under 18 experiencing homelessness. Desirability bias may also have been present in responses surrounding substance use, although there is no evidence to suggest this would differ by SGM status. Finally, our study design was cross-sectional, and therefore could not examine longitudinal trends nor causality between SGM status and our outcomes of interest.

## Conclusion

Our results stand in contrast to previous findings that the overall prevalence of substance use is significantly higher among homeless youth who identify as SGM as compared to heterosexual cisgender homeless youth. Furthermore, our data found that for all substances except methamphetamine, there was no difference in overall burden of negative health outcomes associated with substance use between SGM and heterosexual cisgender youth among our diverse sample of service-seeking youth in San Francisco. These findings may suggest that SGM youth experiencing homelessness who obtain services in a culturally competent setting may experience similar risks from substance use as their heterosexual cisgender peers. While more evidence is needed, future research may support replication of the sexuality- and gender-affirming service model used at Larkin Street: if SGM homeless youth are to build resiliency and overcome the stigma and discrimination that can perpetuate cycles of youth homelessness, culturally-tailored services will be important in addressing substance use and other health issues.

While substance use and mental health outcomes are commonly studied among analyses of homeless youth, our study was unique in its comparison of harmful risks associated with substance use among SGM youth and heterosexual cisgender peers. While research on SGM youth homelessness has expanded in recent years, future research is needed to capture longitudinal data among SGM youth experiencing homelessness, as the fluidity of gender identity and sexuality are not captured by cross-sectional data. Longitudinal studies could thus capture changes in gender identity and sexuality, an especially relevant topic among trans and gender minority youth, and also examine how changes in substance use and mental health may be related to transitions in self-identity and long-term housing stability. Additionally, longitudinal data could help assess the efficacy of various substance use and mental health interventions, especially programs that are tailored for SGM youth experiencing homelessness.

Future research might also examine the intersection of race, SGM identity, and the impact of those and other factors on relative levels of PTSD and depression. Such research, building on the findings presented here, may help to better understand the unique needs and challenges faced by SGM youth experiencing homelessness in order to continue supporting pathways out of homelessness for SGM youth nationwide.
